# Hot Deformation Behaviors of the Mg-3Sn-2Al-1Zn Alloy: Investigation on its Constitutive Equation, Processing Map, and Microstructure

**DOI:** 10.3390/ma13020312

**Published:** 2020-01-09

**Authors:** Yuhang Guo, Yaodong Xuanyuan, Xuannam Ly, Sen Yang

**Affiliations:** 1 School of Materials Science and Engineering, Nanjing University of Science and Technology, Nanjing 210094, China; xuanyuanyaodong@outlook.com (Y.X.); lyxuannam@yahoo.com (X.L.); 2 School of Material Science and Engineering, Jiangsu University of Science and Technology, Zhenjiang 212003, China

**Keywords:** Mg-Sn alloy, hot deformation, constitutive equation, processing maps

## Abstract

In this work, the Mg-3Sn-2Al-1Zn (TAZ321, wt. %) alloy with excellent high temperature resistance was compressed using a Gleeble-3500 thermo-mechanical simulator at a wide temperature and the strain rate range. The kinetics analyses showed that the dominant deformation mechanism was likely caused by the cross slipping of dislocations. A constitutive equation which expressed the relationship between the flow stress, deformation temperature, and strain rate was established, and the average activation energy Q was calculated to be 172.1 kJ/mol. In order to delineate the stability and instability working domains, as well as obtain the optimum hot working parameters of the alloy, the hot processing maps in accordance with Prassad’s criterion are constructed at the true strain of 0.2, 0.4, 0.6, and 0.8, respectively. Based on the hot processing map and microstructure observation, the optimum hot working parameter was determined to be 350 °C/1 s^−1^. The continuous fine dynamic recrystallization (CDRX) grains occurred in the optimum deformation zone. The predicted instability domains was identified as T = 200–300 °C, $\dot{\varepsilon }$ = 10^−2^–1 s^−1^, which corresponded to the microstructure of deformation twinning and micro cracks at the intersection of grain boundaries.

## 1. Introduction

Recently, the Mg-Sn alloy has been considered as a typical heat-resistance magnesium alloy owing to the existence of high melting Mg_2_Sn phases (T_m_–770 °C) [1,2], which have a strong potential for high temperature structural applications. Therefore, it is essential to investigate the hot deformation behaviors in order to design of superior performance Mg-Sn alloys.

To our knowledge, several works have been done on the hot deformation behaviors of Mg-Sn alloys [3,4,5,6]. An example of this is the hot deformation behaviors and optimum processing parameters of homogenized TAZ811 alloy and fine-grained TZA822 alloy investigated by using a processing map and microstructural evolution. The practical processing window of homogenized TAZ811 alloy was determined as T = 350–400 °C and $\dot{\varepsilon }$ = 0.33–3.3 s^−1^, while the dominant high temperature deformation mechanisms were controlled by a climbing of dislocation [6]. In addition, the optimum hot working parameters for the fine-grained TZA822 alloy were determined to be 350 °C/0.01 s^−1^ and 350 °C/10 s^−1^, at which point continuous DRX (CDRX) and discontinuous DRX (DDRX) were the main softening mechanisms [5]. In contrast to the Mg-8Sn based alloys with high Sn content, Mg-3Sn based alloys with a lower amount of Sn did not only decrease the density and solid solution temperature of alloy [7], but also exhibited higher tensile strength and elongation [8]. However, the knowledge on the hot deformation behaviors of the Mg-3Sn alloy in the literature mainly focuses on the Mg-3Sn-1Ca alloy [3,4]. Rao, K.P. et al. reported that there are two stable domains for the as-cast and homogenized Mg-3Sn-1Ca alloy, both of which are in the temperature range 350–550 °C: one is in the lower strain rate range (0.0003–0.01 s^−1^) and the other is found at higher strain rates (1–10 s^−1^) [4].

Compared with the Mg-3Sn-1Ca alloy adopting Ca, the minor addition of Al or Zn as the solid solution strengthening element could enhance the strength [9], age hardening response [10], and strain hardening ability [11] of Mg-Sn based alloys. However, limited research was conducted on the hot deformation behaviors of Mg-3Sn-Al-Zn system alloys. Based on the above consideration, we designed the alloy with nominal composition of Mg-3Sn-2Al-1Zn (wt. %) and investigate the hot deformation behaviors of the alloys in terms of a kinetic analysis, hot processing map, and microstructure evolution. Additionally, hot deformation mechanisms and dynamic recrystallization (DRX) behaviors during the hot deformation process were also analyzed and discussed.

## 2. Experimental Procedure

The alloy ingots with the nominal composition of Mg-3Sn-2Al-1Zn (wt. %) were prepared using an electric furnace with a covering flux under a protective atmosphere (0.5 vol. % SF_6_ + 99.5 vol. % CO_2_). The melt was held at 720 °C for 20 min and mechanically stirred for 2 min to ensure a homogeneous composition, then the pouring was accomplished into a steel die preheated up to 150 °C. After casting, the cast billets were homogenized at 400 °C for 24 h. The actual composition of as-cast and as-homogenized TAZ321 alloy measured using energy dispersive X-ray spectroscopy (EDS, Oxford Instruments Inc, Abingdon, UK) is listed in Table 1. Then the cylindrical samples with a diameter of 10 mm and a height of 15 mm were machined using an electro discharge-cutting machine. Compression tests were conducted on a Gleeble-3500 thermo-mechanical simulator (Dynamic Systems Inc., New York, NY, USA) at the temperature range of 200–350 °C and a strain rate of 10^−3^–1 s^−1^. Mo plates covered with boron nitride powder were used as lubrication for minimizing the effect of friction. Before deformed, the samples were heated at a heating rate of 5 K/s, and followed by a holding time of 10 min for ensuring there were uniform temperature throughout the samples. In order to freeze the microstructure, all post-deformation samples were cooled by water-quench up to a true strain of 0.8. The microstructural examinations specimens were sliced in the center parallel to the compression axis.

The phase structure of as-homogenized TAZ321 alloy was identified by Parnike X’ Pert PRO MPD (Holland Panalytical, Almelo, The Netherlands) X-ray diffraction (XRD) diffractometer with Cu radiation. The microstructure of the as-homogenized and post-deformation samples were examined using an AxioImager A2m (ZEISS Inc., Oberkochen, Germany) optical microscope (OM), a MALA3 Triglay (TESCAN Inc., Brno, Czech) scanning electron microscope (SEM) equipped with EDS, and a 2100 F (JEOL Inc., Tokyo, Japan) transmission electron microscope (TEM). Additionally, the electron backscatter diffraction (EBSD) micro orientation analysis of the as-homogenized and post-deformation samples was conducted using an Oxford Instruments-NordlysNano EBSD detector (Oxford Instruments Inc., Abingdon, UK) equipped on the SEM operating at 20 kV. The samples prepared for EBSD analysis were additionally developed by ion etching in a Leica RES101 ion etching apparatus (Bal-Tec, Inc., Wetzlar, Germany) after mechanical polishing. The orientation imaging microscopy software used for post-EBSD data analysis was provided by HKL Channel 5.11.20405.0, Oxford Instruments NanoAnalysis, Abingdon, UK.

## 3. Results and Discussion

### 3.1. Microstructural Characterization and Analyze of the as-Homogenized TAZ321 Alloy

**Figure 1:**
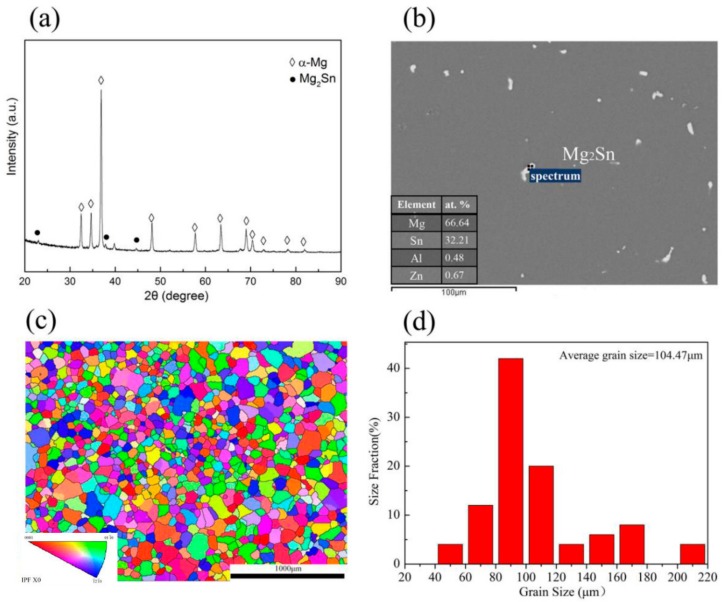
The microstructure of the as-homogenized TAZ321 alloy: (**a**) XRD; (**b**) SEM and EDS; (**c**) Inverse pole map; (**d**) Grain distribution statistic.

Figure 1 shows the initial microstructure of the as-homogenized TAZ321 alloy. XRD results (Figure 1a) exhibit that the alloy is mainly composed of α-Mg phases, and some weak peaks representing Mg_2_Sn phases can also be observed. Based on the SEM and EDS results (Figure 1b), the undissolved spherical and/or cubic Mg_2_Sn phases distribute homogeneously into the α-Mg matrix. An EBSD inverse pole map (IPF) (Figure 1c) and grain distribution statistic (Figure 1d) show that the grain size ranged from 40 to 200 μm with random grain orientations, and the average grain size was determined to be 104.47 μm.

### 3.2. Flow Stress Curves Analysis

**Figure 2:**
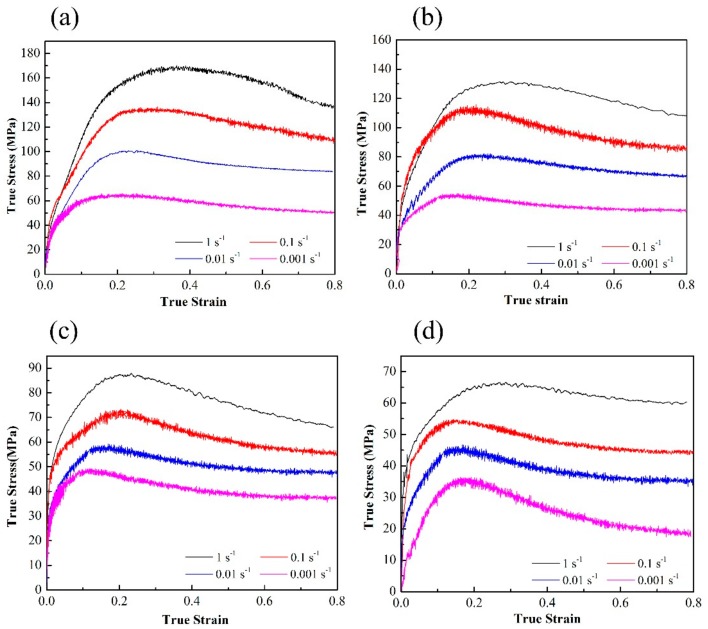
True stress-true strain curves of as-homogenized TAZ321 alloy at different strain rates with temperature: (**a**) 200 °C; (**b**) 250 °C; (**c**) 300 °C; (**d**) 350 °C.

The true stress-strain curves of the homogenized TAZ321 alloy under different temperature and strain rates at a true strain of 0.8 are indicated in Figure 2. The flow curves initially increase to a peak value and then decrease and finally reach a steady state. Such flow behavior is a typical characteristic for hot working accompanied by work hardening and dynamic softening. The work hardening was induced generally by the generation and multiplication of dislocation, while the dynamic softening was related to the DRX and/or DRV [12]. In addition, the flow stress decreased with the decreasing of the strain rate, which was attributed to a more adequate time for the mobility of grain boundaries and dislocation at a lower strain rate.

**Figure 3:**
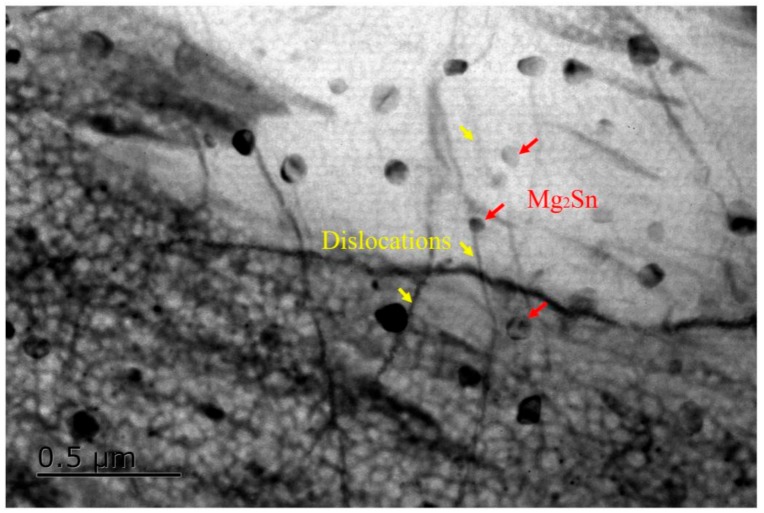
The interaction effect between dislocations and Mg_2_Sn particles of the homogenized TAZ321 alloy deformed at 200 °C/10^−3^ s^−1^.

Furthermore, it was interestingly found that obvious serration behaviors can be observed in the true stress-strain curves. The type of serrations was associated with the deformation temperature and strain rate, as well as the extent of deformation, which can be attributed to the interaction effect between the dislocation and precipitation and/or solute atoms, i.e., the dynamic strain aging (DSA) effect [13,14]. It is thought to be when a dislocation movement is hindered by obstacles, solute atoms will segregate preferentially by means of pipe diffusion in the vicinity of the obstacle, resulting in the formation of solute cluster, which can strongly pin the dislocation, leading to the stress increase. Meanwhile, the pinning dislocation will be released due to thermal activation and then cause a stress decrease. Thus, serrated behaviors during deformation can be understood as the circulation of pinning and releasing of mobile dislocations. Based on the serration morphologies, the serration type can be categorized. Type A is manifested by the stress sudden rise and then by dropping to a general level at a random frequency. Type B is also called the “hopping band”, and is characterized by the appearance of oscillation above and below the average value of stress in quick succession, and type C is defined as stress fluctuation with a higher amplitude and frequency in comparison with type B [14]. It should be noted that the serrations morphology is indicative for type B for the TAZ321 alloy, which was attributed to the interaction of the dislocation and the dynamic precipitation of Mg_2_Sn particles, as illustrated in Figure 3.

### 3.3. Constitutive Analysis

For the metal materials, there are three different constitutive models (power, exponential, or hyperbolic sine law) can be used to express the relationship among the strain rate ($\dot{\varepsilon }$), deformation temperature (T), and flow stress (σ) for different deformation conditions. The power, exponential or hyperbolic sine law can be described by Equations (1)–(3), respectively [15]:

(1) $$\dot{\varepsilon }=A_{1}\sigma^{n_{1}}\;\left(\alpha \sigma <0.8\right)$$

(2) $$\dot{\varepsilon }=A_{2}\exp\left(\beta \sigma \right)\;\left(\alpha \sigma >1.2\right)$$

(3) $$Z=\dot{\varepsilon }\exp\left(\frac{Q}{\mathrm{RT}}\right)=A{\left[\sin h\left(\alpha \sigma \right)\right]}^{n}\;\;\;\left(\mathrm{universal}\right)$$

where Z is the Zener-Hollomon parameter, A, A_1_, A_2_, n_1,_ and β are the material constants. α is the stress multiplier, n is the stress exponent, Q is the activation energy of hot deformation (kJ mol^−1^), and R is the gas constant (8.314 J mol^−1^ K^−1^). Taking natural logarithm simultaneously on both sides of Equations (1)–(3), the following equations were obtained, namely:

(4) $$\ln\;\dot{\varepsilon }\;={\mathrm{lnA}}_{2}+\;\beta \sigma -\frac{Q}{\mathrm{RT}}$$

(5) $$\ln\;\dot{\varepsilon }\;={\mathrm{lnA}}_{1}{+n}_{1}\ln\sigma -\;\frac{Q}{\mathrm{RT}}$$

(6) $$\mathrm{lnZ}=\ln\;\dot{\varepsilon }\;+\frac{Q}{\mathrm{RT}}\;=\;\mathrm{lnA}+\;n\;\ln\left[\sinh\left(\alpha \sigma \right)\right].$$

In general, the power law was adopted for the low stress level (ασ < 0.8), the exponential law was suitable at a high stress level (ασ > 1.2), while the sine hyperbolic law combined the power law and the exponential law to break the stress limits and can be considered as a suitable candidate for wide deformation conditions. Therefore, the sine hyperbolic law model was employed for the homogenized TAZ321 alloy.

**Figure 4:**
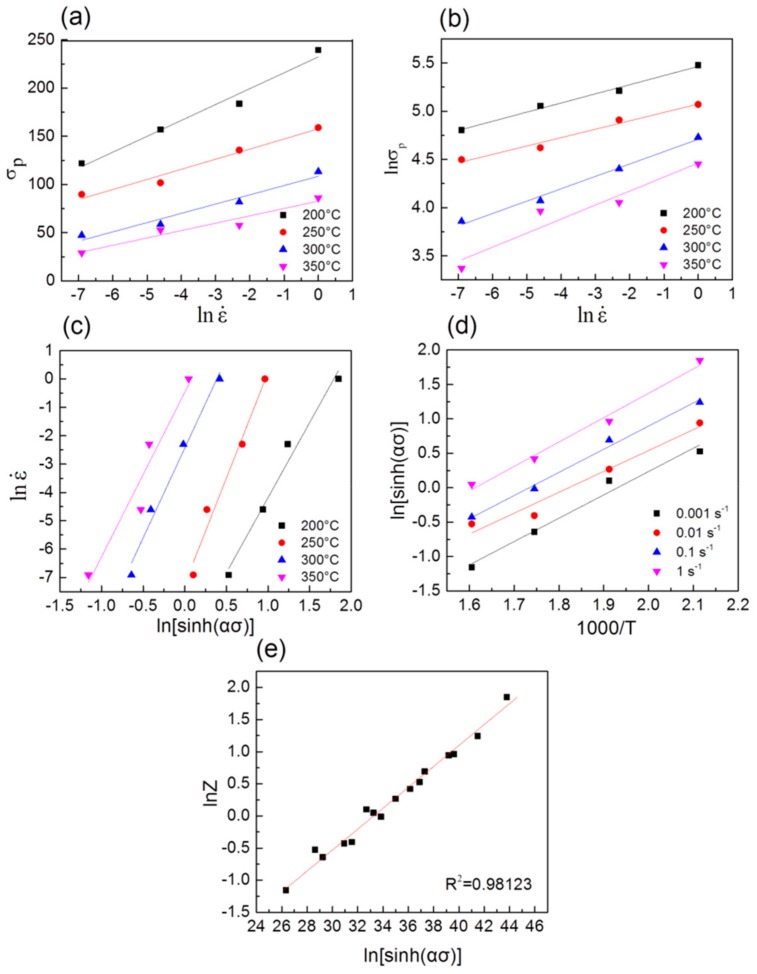
Relationship between (**a**) ${\ln\;\dot{\varepsilon }\;–\sigma }_{p}$, (**b**) $\ln\;\dot{\varepsilon }–{\ln\sigma }_{p}$, (**c**) $\ln\;\dot{\varepsilon }–\ln\sin h\left(\sigma_{p}\right)$, (**d**) $\ln\sin h\left(\sigma_{p}\right)–\frac{1000}{T}$, and (**e**) $\mathrm{lnZ}\;–\ln\left[\sinh\left(\alpha \sigma \right)\right]$, respectively.

By linear fitting the relationship of $\ln\;\dot{\varepsilon }\;–\sigma$ and $\ln\;\dot{\varepsilon }\;–\ln\sigma$ in Figure 4a,b, the average values of n_1_ and β, i.e., the linear slope of the plots, can be obtained as 9.1859 and 0.01064. Thus, the value of α can be calculated as 0.001158. After submitting the value of α into Equation (6), the linear relationship can be determined (Figure 4c). The average value of stress exponent was equal to the slope of ${\ln\;\dot{\varepsilon }\;–\ln\sinh\sigma }_{P}$ and was obtained as 6.22. For the Mg alloy, the value of stress exponent n represents the hot deformation mechanisms during the process of hot deformation. The range of this exponent was n = 4–6 during the dislocation climb, and while the value of n ranged from 6 to 7, the cross-slip of screw dislocations were the main deformation mechanisms [5,16]. For the present as-homogenized TAZ321 alloy, the average value of n was close to that of fine-grained TZA822 alloy (n = 6.45), indicating that the dominant deformation mechanism was likely controlled by dislocation cross-slip.

In addition, by taking partial differential on both sides of Equation (4), the activation energy Q associated with diffusion and the precipitation of particles could be conveyed as:

(7) $${\;Q}_{\mathrm{eff}}=R\cdot S\cdot N=R\cdot {\left\{\frac{\partial \left(\ln\;\dot{\varepsilon }\right)}{\partial \ln\left[\sinh\left(\alpha \left(\sigma_{p}{-\sigma }_{0}\right)\right)\right]}\right\}}_{T}\cdot {\left\{\frac{\partial \ln\left[\sinh\left(\alpha \left(\sigma_{p}{-\sigma }_{0}\right)\right)\right]}{\partial \left(\frac{1000}{T}\right)}\right\}}_{\;\dot{\varepsilon }}.$$

The value of S was equal to the slope of the plots of $\ln\;\sinh\left(\sigma_{p}\right)–\;\frac{1000}{T}$, as shown in Figure 4d. The average value of Q of the alloy was calculated as 172.1 kJ/mol, which was slightly lower than the activation energy of homogenized TAZ811 alloy (−187.2 kJ/mol) [6] and fine-grained TZA822 alloy (−189.5 kJ/mol) [5], and was higher than that of AZ61 alloy (−140 kJ/mol) [17] and Mg-3Zn-0.8Zr alloy (−124.6 kJ/mol) [18]. The higher Q value indicated the existence of desperate Mg_2_Sn particles, which can provide back stress to hinder the movement of dislocation, resulting in higher Q values.

Moreover, the corresponding value of A and n could be determined as $2.78\times 10^{14}$ and 6.14 by the regression analysis of the intercept of plots of $\ln Z–\ln\left[\sinh\left(\alpha \sigma \right)\right]$ based on Equation (6), as indicated in Figure 4e. The correlation coefficient (R^2^ = 0.9892) was almost 1, validating that the hyperbolic sine model matched well with the experiment data. Finally, substituting the corresponding parameters into the Equation (1), the constitutive equation of the homogenized TAZ321 alloy was obtained as follows:

(8) $$\dot{\varepsilon }=2.78\times 10^{14}\cdot {\left[\sinh\left(0.0012\sigma \right)\right]}^{6.14}\cdot \exp\left(-\frac{172110}{8.314T}\right).$$

### 3.4. Processing Maps

Materials engineers usually get the optimum working parameters of the alloys by utilizing the hot processing map on the basis of the dynamic materials model (DMM), i.e., Prassad’s criterion [19]. It was proven to be an effective tool for understanding the hot deformation behaviors of metallic alloys, such as titanium alloys [20,21], twinning induced plasticity (TWIP) steels [22] and super-alloys [23].

The hot processing maps were overlapped by the power dissipation map and instability map based on the DMM model, and the overall total power P was considered as the sum of G and J. G is the power dissipation value caused by plastic deformation, and J is power dissipation related to the microstructural evolution, such as during phase transformation, DRV, and DRX. The allocation of P for the G and J was determined by a strain rate sensitivity parameter m, which can be expressed by the formula as [24]:

(9) $$m\;=\;\frac{\partial J}{\partial G}\;=\;\frac{\partial \left(\ln\sigma \right)}{\partial \left(\ln\;\dot{\varepsilon }\right)}.$$

In addition, for a non-linear power dissipater, the capacity of power dissipation was expressed by means of a power dissipation (η) equation given by [24]:

(10) $$\eta =\frac{2m}{2m\;+\;1}$$

where m is the strain rate sensitivity. The flow instability region can be predicted by the instability parameter $\xi \left(\dot{\varepsilon }\right)$, which is given by Equation (11) [24]:

(11) $$\xi (\dot{\varepsilon })\;=\;\frac{\partial \mathrm{lg}\left(\frac{m}{m\;+\;1}\right)}{\partial \mathrm{lg}\;\dot{\varepsilon }}\;+\;m\;\leq \;0.$$

When the value of $\xi \left(\dot{\varepsilon }\right)$ is negative, flow instability regions occur in the deformed samples, which are characterized by the presence of cracks, local flow, and adiabatic shear band in the microstructure [25].

**Figure 5:**
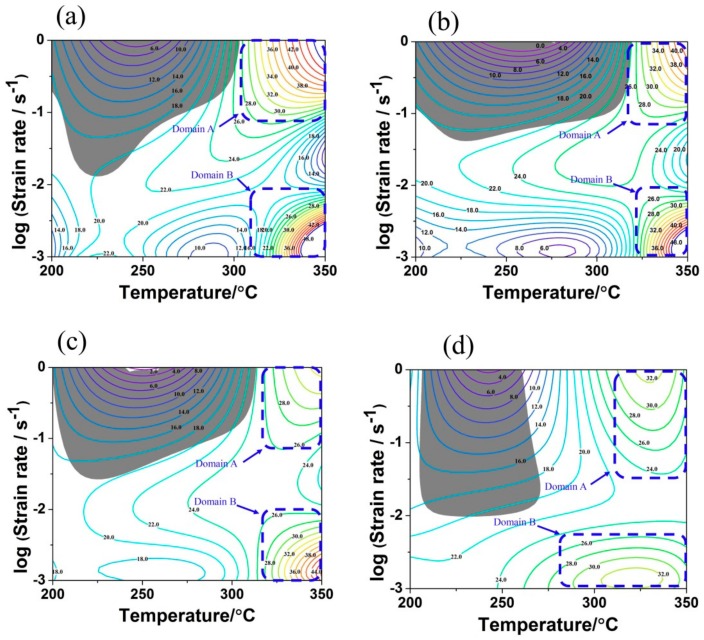
The hot processing maps of homogenized TAZ321 alloy at the true strain of (**a**) 0.2, (**b**) 0.4, (**c**) 0.6, and (**d**) 0.8.

Figure 5 shows the hot processing maps of the as-homogenized TAZ321 alloy at different true strain, in which the contour represents the power dissipation efficiency as a percentage and the gray shadow regions denote the regimes of flow instability. There are two common stability domains (domain A and domain B) with the relatively higher power efficiency at different true strain levels. Domain A is located at T = 310–350 °C and $\dot{\varepsilon }$ = 10^−^^1^–1 s^−^^1^, corresponding to the value of activation range from 160.5 kJ/mol to 176.7 kJ/mol. Domain B is observed at T = 310–350 °C, $\dot{\varepsilon }$ = 10^−3^–10^−2^ s^−1^, corresponding to a value of activation which ranged from 146.2 kJ/mol to 178.1 kJ/mol. In addition, the area fraction of instability domains decreased with the increasing true strain. Commonly, domains with the high η value in stability regions are treated as the ideal processing windows, which also need to further confirmed by the microstructures of deformed samples [6,12].

**Figure 6:**
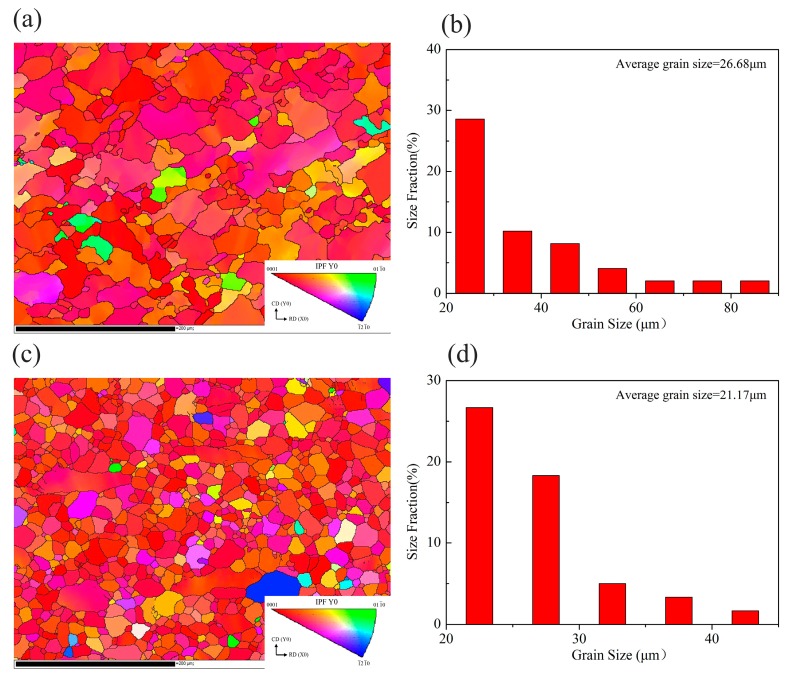
Inverse pole Figures (IPF) and grain distribution statistics of the samples deformation at (**a**,**b**) 350 °C/10^−3^ s^−1^; (**c**,**d**) 350 °C/1 s^−1^. (In the IPF, the CD and RD refer to the compressive direction and rolling direction, respectively.).

Figure 6a,c shows the EBSD maps of the samples deformed at 350 °C/10^−3^ s^−1^ and 350 °C/1 s^−1^, corresponding to the domain B and A with the high η values in the processing map, respectively. Compared to the as-homogenized alloy, the post-deformation samples at 350 °C/10^−3^ s^−1^ exhibit a smaller grain size with an average grain size of 26.68 μm, which was attributed to the appearance of DRX. With the strain rate increased to 1 s^−1^, the DRX grains become more homogeneous and the average grain size decreased to 21.17 μm. This was due to DRX, which plays an important role in refining microstructure and improving mechanical properties [26]. Thus, based on the hot process map and microstructure, the domain A with homogeneous DRX grains can be determined as an optimum workability domain for the as-homogenized TAZ321 alloy.

**Figure 7:**
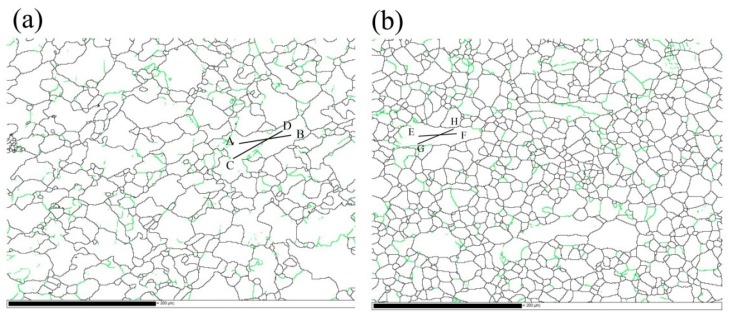
The grain boundary (GB) distribution maps of homogenized TAZ321 alloy at (**a**) 350 °C/10^−3^ s^−1^; and (**b**) 350 °C/1 s^−1^ (grain boundaries from 2 to 15° are in green and high angle boundaries that are >15° are in black).

**Figure 8:**
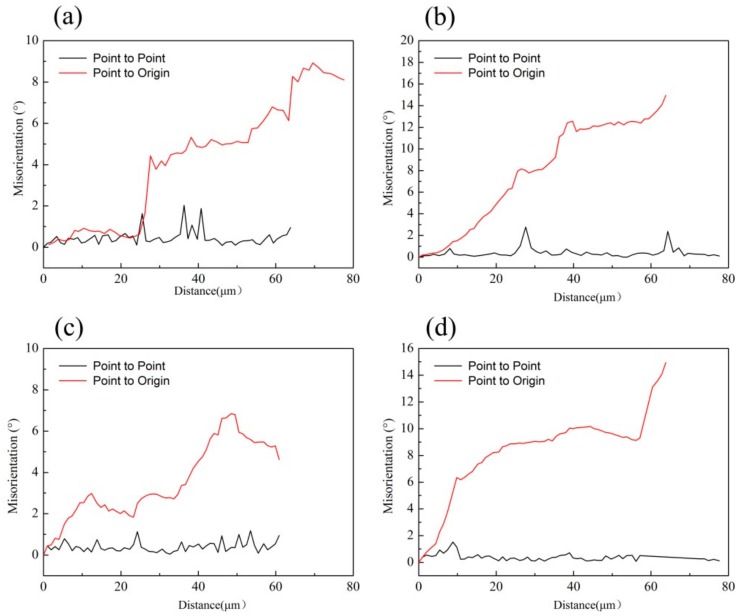
Misorientations measured along the lines of (**a**) AB, (**b**) CD, (**c**) EF, (**d**) GH.

For the DRX, it is commonly believed that discontinuous DRX (DDRX) and continuous DRX (CDRX) are the main two mechanisms. Bulging original grain boundaries are the domain nucleation sites of DDRX. Meanwhile, the CDRX mechanism is characterized as the continuous absorption of dislocations in low-angle grain boundaries (subgrain boundaries) and followed by the progressive rotation of subgrains, which leads to the formation of high-angle grain boundaries (HAGBs) [5,6]. Figure 7a,b shows the GB maps of the post samples deformed at 350 °C/10^−3^ s^−1^ and 350 °C/1 s^−1^, respectively. As indicated, no obvious grain boundaries bulging was observed and the formation of subgrains (grain boundaries from 2 to 10°) signifies the occurrence of continuous DRX (CDRX) in the alloy. Furthermore, the subgrain evolution can be evaluated by the changes of local misorientaion (point to point) and cumulative misorientaion (point to origin) [27]. Figure 8a–d indicate the location misorientation (black lines) and cumulative misorientation (red line) along the AB, CD, EF, and GH lines, respectively. From Figure 8, it can be found that the values of local misorientation along the grain boundaries and in the grain interior did not extend beyond 5 degrees, while the cumulative misorientations are high, indicating a large increase in misorientation. Adequate misorientations can promote the subgrain rotation needed to form HAGBs.

**Figure 9:**
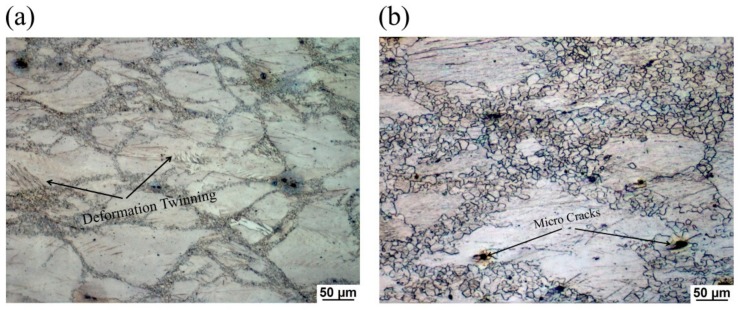
OM images of homogenized TAZ321 alloy deformed at the instability regions: (**a**) 200 °C/10^−2^ s^−1^, (**b**) 250 °C/10^−1^ s^−1^.

The instability regions predicted by the processing map are mainly located at the median temperature regions, which were identified as T = 200–300 °C, $\dot{\varepsilon }$ = 10^−2^–1 s^−1^. Figure 9a,b shows the OM images of the specimens compressed at 200 °C/10^−2^ s^−1^ and 250 °C/10^−1^ s^−1^ at a true strain of 0.8, respectively, corresponding to the microstructure of instability domains in the processing map. The microstructure of the samples deformed at the instability regions was characterized by deformation twining at the interior of grains and micro cracks along the intersection of grain boundaries, which were considered to be induced by flow localization. This was because deformation twining can induce local stress concentration and micro cracks can lead directly to fracture, which should be avoided during hot deformation [5].

## 4. Conclusions

(1) The flow curves exhibited initially increase to a peak value and then decrease and finally reach a steady state. The serrations morphology of the flow curves is indicative for type B for the TAZ321 alloy, which was attributed to the interaction between the dislocations and the dynamic precipitation of Mg_2_Sn particles.
(2) The relationship between the flow stress, deformation temperature, and strain rate of homogenized TAZ321 alloy could be described by the constitutive equation as follows: $\dot{\varepsilon }\;=\;2{.78\;\times \;10}^{14}\cdot {\left[\sinh\left(0.0012\sigma \right)\right]}^{6.14}\cdot \exp\left(-\frac{172110}{8.314T}\right)$ $\dot{\varepsilon }=4.75\times 10^{11}{\left[\sinh\left(0.004\sigma \right)\right]}^{4.11}\exp\left(-\frac{322300}{8.314T}\right)$, the stress exponent n being 6.22, suggesting that the dominant deformation mechanism was likely to be controlled by the cross-slipping of dislocation.
(3) According to the processing map, there were two stability domains with relatively high power dissipation efficiency: one was representing domain A (T = 310–350 °C, $\dot{\varepsilon }$ = 10^−^^1^–1 s^−^^1^), and the other was denoted as domain B (T = 310–350 °C and $\dot{\varepsilon }$ = 10^−3^–10^−2^ s^−1^). The optimum hot working domains were determined as being part of domain A (T = 310–350 °C and $\dot{\varepsilon }$ = 10^−1^–1 s^−1^) in combination with the hot processing map and microstructure observation, which corresponded to the microstructure of fine CDRX grains.
(4) The instability regions predicted by the processing map were identified as T = 200–300 °C, $\dot{\varepsilon }$ = 10^−2^–1 s^−1^. Deformation twinning or micro-cracks at the intersection of grain boundaries were observed in the instability domains.
(5) The TAZ321 alloy deformed at 350 °C/1 s^−1^ exhibited more a homogeneous and finer grain size, and therefore were viewed strongly as a candidate for structural application.

## Figures and Tables

**Table 1:** Actual composition (wt. %) of the as-cast and as-homogenized TAZ321 alloy.

Alloy	Mg	Sn	Al	Zn
As-cast	93.87 ± 0.46	3.45 ± 0.21	1.64 ± 0.18	1.05 ± 0.07
As-homogenized	93.50 ± 0.26	3.47 ± 0.3	1.8 ± 0.23	1.23 ± 0.32
